# Exogenous Uniconazole promotes physiological metabolism and grain yield of rice under salt stress

**DOI:** 10.3389/fpls.2024.1459121

**Published:** 2024-09-19

**Authors:** Xiaole Du, Youwei Du, Naijie Feng, Dianfeng Zheng, Hang Zhou, Jingxin Huo

**Affiliations:** ^1^ College of Coastal Agricultural Sciences, Guangdong Ocean University, Zhanjiang, China; ^2^ National Saline-tolerant Rice Technology Innovation Center, South China, Zhanjiang, China; ^3^ Shenzhen Institute of Guangdong Ocean University, Shenzhen, China

**Keywords:** salt stress, rice, uniconazole, physiological, yield

## Abstract

**Introduction:**

Salt stress severely inhibit plant growth and development. Uniconazole has been considered to significantly increase plant stress tolerance. However, the mechanism by which Uniconazole induces salt tolerance in rice seedlings and its impact on yield is still unclear.

**Methods:**

In this study, the effects of exogenous Uniconazole on morphogenesis, physiological metabolism, and yield of rice seedlings under salt stress were analyzed using the salt-tolerant rice variety HD961 and the salt-sensitive rice variety 9311.

**Results:**

The results showed that salt stress significantly inhibited rice growth, disrupted the antioxidant system and pigment accumulation, and reduced photosynthesis, and yield. There were corresponding percent decreases of 13.0% and 24.1% in plant height, 31.6% and 55.8% in leaf area, 65.7% and 85.3% in root volume, respectively for HD961 and 9311. spraying However, compared to salt stress, the US treatment increased the percentage to 4.7% and 139.0% in root volume, 7.5% and 38.0% in total chlorophyll, 4.5% and 14.3% in peroxidase (POD) of leaves, 14.4% and 54.2% in POD of roots, 18.7% and 22.7% in catalase (CAT) of leaves, and 22.6% and 53.9% in CAT of roots, respectively, for HD961 and 9311. In addition, it also significantly enhanced photosynthesis at the reproductive stage, promoted the transport of carbohydrate to grains. And US treatment significantly increased the percentage to 9.0% in panicle length, 28.0% in panicle number per hole, 24.0% in filled grain number, 3.0% in 1000-grain weight, and 26.0% in yield per plant, respectively, for HD961, compared to salt stress.

**Discussion:**

In summary, applying Uniconazole at the seedling stage can alleviate the damage induced by NaCl stress on rice by regulating the physiological metabolism of rice plants. This reduces the negative effects of salt stress, enhance salt tolerance, and boost rice production.

## 1 Introduction

Soil salinization is a major limiting factor in world agricultural production, caused mostly by inadequate drainage, poor irrigation systems, climate change (resulting in rising sea levels), and drought (Rengasamy, 2006). Globally, abiotic stresses, such as salinity are a key obstacle to crop production in planting areas (Zhang et al., 2017). Soil salinity has covered 20% of the world’s cropland and 33% of irrigated farmland (Shrivastava and Kumar, 2015). It is estimated that salt stress reduces global crop yield by about 20%, resulting in a loss of at least USD 1.2 billion per year (Alkharabsheh et al., 2021). As a result, improving the salt tolerance and yield of crops is not only contributes to the effective utilization of saline-alkali land but also supports sustainable agriculture and mitigating the world food crisis.

Rice (*Oryza sativa* L.) is the staple food consumed by more than half of the world’s population, especially in Asia and Africa. It is a valuable food crop that provides food security for most countries around the world (Majumder et al., 2019). Rice is a salt-sensitive crop (Parihar et al., 2015; Roy et al., 2014), high salt concentrations inhibit root growth and development by reducing water and nutrient uptake by roots. It accelerates the decomposition of chlorophyll, leading to a decrease in the photosynthetic rate, which in turn has an inhibitory effect on growth of rice shoots (Gadelha et al., 2021), resulting in a decline in grain yield (Nasrudin et al., 2022). Salt stress can cause ion imbalance and ion toxicity, impairing the photosynthetic system (Farooq et al., 2015), reduce the efficiency of electron transfer, and the generation of superoxide anion (O_2_^·-^), while O_2_^·-^ can cause excessive production of reactive oxygen species (ROS), leading to oxidative damage in plant cells. This damage can result in a decreased enzyme activity and increased membrane lipid peroxidation. In severe cases, it can react with important biological molecules like lipids, proteins, and nucleic acids, thereby inhibiting plant growth and development and leading to cell death (Mittler, 2002). Rice has been reported to be more sensitive to salt stress in the early vegetative stage as well as in the later reproductive stage (Ismail and Horie, 2017). Therefore, improving the salt resistance of rice seedlings is an important measure to ensure seedling growth and increase yield in the later stages. Although some research has shown that rice can be genetically engineered to be more salt-tolerant (Raza et al., 2023), many European and non-European countries consider this is less environmentally beneficial (Eckerstorfer et al., 2019). However, previous studies have shown that exogenously applied plant growth regulators can effectively improve plants’ salt tolerance (Khan et al., 2021).

Uniconazole is a triazole plant growth retardant with high efficiency, low toxicity, and low residue (Fletcher et al., 1999), which can improve crop yield and quality. Many studies have shown that uniconazole can improve the resistance of crops to abiotic stresses, such as high temperature (Zhou and Leul, 1999), low temperature (Hu et al., 2022), flooding (Wang et al., 2022; Qiu et al., 2005), drought (Keshavarz and Khodabin, 2019), and salinity stress (Al-Rumaih, 2007). It has been extensively utilized in crops such as soybean (Zhou et al., 2021), maize (Ahmad et al., 2021), and rapeseed (Zuo et al., 2020). Spraying uniconazole on mung beans increased the activity of antioxidant enzymes such as ascorbate peroxidase (APX) and peroxidase (POD) and net photosynthetic rate. This further promoted the expression levels of photosynthetic genes, enhanced cold tolerance, and increased mung bean yield (Yu et al., 2022). Foliar application of Uniconazole to rapeseed improves its tolerance to high temperatures and other stresses in rapeseed (Zhou and Leul, 1999). It also has enhanced drought tolerance in rice (Zeng et al., 1994). Recent studies have shown that Uniconazole can increase maize yield by altering maize ear shape, photosynthetic efficiency, and antioxidants under salt stress (Xu et al., 2022). Previous studies have shown that uniconazole can reduce lodging rates and alleviate plant damage under stress. Therefore, we speculated that Uniconazole could improve rice resistance to salt stress by modulating the morphological and physiological metabolism.

No study has systematically examined the effect of the plant growth regulator Uniconazole treatment on rice plants under salt stress. The goal of this study was to investigate the influence of the exogenous plant growth inhibitor Uniconazole on seedling morphology, chlorophyll content, photosynthetic indices, physiology, and yield in salt-stressed rice. This study adds to our knowledge of the regulatory effects of using Uniconazole on rice seedling development and offers novel perspectives on how uniconazole regulates rice yield and salt tolerance.

## 2 Materials and methods

### 2.1 Plant material and growth condition

Different genotypes of Indica rice cultivars HD961 (salt-tolerant, red rice) and 9311 (salt-sensitive, white rice) were used as experimental materials, which were acquired from the Germplasm Resource Bank of Coastal Agricultural College at Guangdong Ocean University(Zhanjiang, chain). HD961 and 9311 have been used in abiotic stress-related studies (Yang et al., 2022). At the seedling stage (from germination to the four leaves and one heart), the cultivation substrate consisted of a mixture of latosol (dried and sieved) with sand at a ratio of 3:1 (V:V), with 2.5 kg per pot (upper base diameter: 19 cm, lower base diameter: 13 cm, height: 16.5 cm). The cultivation substrate was air-dried latosol at the large seedling stage (from the four leaves and one heart stage to full maturity stage), with 8.5 kg per barrel (diameter: 30 cm, height: 22 cm).

### 2.2 Experimental design

The study was conducted in 2022 at the Coastal Agriculture College’s greenhouse, Guangdong Ocean University (Zhanjiang, China), under controlled conditions (natural light, day/night temperature of 25/20 ± 2°C, and 60% relative humidity) using potted plants. Healthy, full, and whole HD961 and 9311 rice seeds were selected and sterilized with 3% H_2_O_2_ for 10 min, rinsed with distilled water 3-5 times, and the residual H_2_O_2_ was thoroughly washed. Seeds were soaked in distilled water and germinated for 24 h at 30°C in the dark before sowing. In total, 1 L of fertilizer solution (urea: 0.146 g·L^-1^, potassium chloride: 0.125 g·L^-1^, diammonium phosphate: 0.200 g·L^-1^) was watered on the pot the day before sowing. The rice seeds with the same white dew were sown in a nutrient bowl, and 69 seeds were evenly sown in each pot, with a plant spacing of 1 cm.

Using a completely random design, two rice varieties were set up with four treatments, each with 10 replicates. Plants were grown to the one-leaf-one-heart stage (6 days after sowing) and subjected to treatment with Uniconazole. Distilled water or a solution containing 10 mg·L^-1^ of Uniconazole (a small amount of No. 8 additives and alcohol should be used to aid in dissolution) and clear water was sprayed using a hand-held sprayer around 18:00 on a sunny afternoon (spray the front and back of each leaf evenly to moisten them without causing dripping). Sprayed only once during the test. After 24 h of uniconazole treatment, the corresponding treatments with NaCl and distilled water were conducted. The experimental treatments were as follows: (1) CK: 0% (0 mmol·L^-1^) NaCl + Spraying water; (2) U: 0% (0 mmol·L^-1^) NaCl + Spraying 10 mg·L^-1^ Uniconazole; (3) S: 0.6% (102.56 mmol·L^-1^) NaCl + Spraying water; (4) US: 0.6% (102.56 mmol·L^-1^) NaCl + Spraying 10 mg·L^-1^ Uniconazole. The 0.6% NaCl solution or clear water was replenished at 2-day intervals with three times the amount of water to maintain concentration. Samples were collected from four leaves and one heart stage and quickly frozen in liquid nitrogen and stored in a refrigerator at -40°C for further testing.

Each plastic barrel was irrigated with 4 L of either clear water or salt water two days before the rice seedlings were transplanted. After the water surface was stable, marking was made, and then water was regularly added to keep the water layer (2 cm). At the same time, the base fertilizer (urea: 0.499 g, diammonium phosphate: 0.445 g, potassium chloride: 0.712 g) was applied and stirred evenly. When the seedlings have grown to four leaves and one heart stage, the seedlings were selected with consistent growth and were transplanted into large plastic barrel. Each barrel has three holes, and two plants was in one hole. Tillering fertilization (urea: 0.552 g) was applied one week after transplanting, and panicle fertilizer (urea: 0.552 g, potassium chloride: 0.182 g) was applied when the second leaf had expanded by 1/3. Each treatment was repeated 12 times using a completely random placement design, and the yield indicators were measured after reaching full maturity.

### 2.3 Indicators and methods of determination

#### 2.3.1 Determination of seedling growth index

After rinsing the rice seedlings from each treatment with clean water, 20 representative seedlings were selected from each pot. Plant height, stem diameter, and leaf area of each individual rice seedling were measured by slide gauge (0.1 mm) and leaf area meter (0.1 mm^2^). The shoots and roots fresh weight were measured using an electronic balance (0.0001 g) after the excess water was absorbed by filter paper. Subsequently, the rice seedlings were dried at 105°C for 30 minutes and dried to a constant weight at 75°C, and the shoot dry weight and root dry weight were measured.

#### 2.3.2 Determination of root morphological index

In each treatment, 10 seedlings with the same growth vigor were selected and rinsed with water until there was no soil. Then they were placed in a glass sink with water to adjust the root position. The root system was scanned using Win RHIZO LA6400 XL to capture images. These images were then analyzed using the accompanying root analysis software (Win RHIZO Pro)to obtain measurements such as total root length, root surface area, root volume and average root diameter.

#### 2.3.3 Determination of chlorophyll content

Chlorophyll a (Chl a), chlorophyll b (Chl b), carotenoid (Car), and total chlorophyll (Chl a + b) were determined using the method proposed by Kolomeichuk (Kolomeichuk et al., 2020). Fresh leaves (0.1 g) were soaked in 10 ml 95% ethanol in the dark for 24 hours (leaves completely faded). The concentrations of Chl a, Chl b, and Car were measured by spectrophotometry at 665, 649, and 470 nm, respectively.


$$\text{Chl a}\left(\text{mg}\cdot {\text{g}}^{-1}\right)=13.95\;{\text{A}}_{665}-6.88\;{\text{A}}_{649},$$

$$\text{Chl b}\left(\text{mg}\cdot {\text{g}}^{-1}\right)=24.96\;{\text{A}}_{649}-7.32\;{\text{A}}_{665},$$

$$\text{Car }\left(\text{mg}\cdot {\text{g}}^{-1}\right)=\left(1000\;{\text{A}}_{470}-2.05\text{ Chl a}-114.8\text{ Chl b}\right)/245,$$

$$\text{Total Chl }\left(\text{mg}\cdot {\text{g}}^{-1}\right)=\text{Chl a}+\text{Chl b}.$$

#### 2.3.4 Determination of photosynthetic index

Photosynthetic parameters – including the net photosynthetic rate (*P_n_*), stomatal conductance (*G_s_*), intercellular carbon dioxide concentration (*C_i_*), and transpiration rate (*T_r_*) were measured were measured at 8:30 - 11:00 a.m. with a Li-6800 photosynthesis instrument equipped with an LED leaf chamber (Li-Cor Inc., Lincoln, USA). Five plants were measured in each treatment. The middle part of the fully expanded sword-leaf of rice was measured at both the booting stage and the full heading stage.

#### 2.3.5 Physiological index determination

Peroxidase (POD) was determined using the guaiacol method, while catalase (CAT) and ascorbate peroxidase (APX) were determined using spectrophotometry.

Determination of POD Content: In the presence of hydrogen peroxide, peroxidase catalyzes guaiacol to synthesize brownish red product-tetra-o-methoxyphenol (polymer). At 470 nm, 0.3 mL of 0.2 M phosphate buffer (pH 6.0), 0.03 mL of 30% H_2_O_2_, and 0.02 mL of 0.05 M guaiacol were added to 10 μL of enzyme solution to initiate the reaction. The activity of the enzyme is expressed as the change in absorbance per minute (Cakmak and Marschner, 1992).

Determination of CAT Content: Hydrogen peroxide has a strong absorption at 240 nm ultraviolet light. Catalase catalyzes the decomposition of hydrogen peroxide. In the reaction system, 0.1 mL of enzyme solution (1.9 mL of 50 mM phosphate buffer (pH 7.0) and 1 mL of 0.075% H_2_O_2_ to initiate the reaction. The activity of the enzyme was expressed as a decrease in absorbance at 240 nm per minute (Fu and Huang, 2001).

Determination of APX Content: Ascorbate Peroxidase (APX) catalyzes the oxidation of ascorbic acid (ASA) by hydrogen peroxide. ASA has the maximum absorption at 290 nm. The reaction system consisted of:1 mL of 50 mM phosphate buffer (pH 7.0), 2 mM ethylenediaminetetraacetic acid, 1 mL of 0.3 mM ascorbic acid, and 20 μL of 30% H_2_O_2_. 0.1 mL of enzyme solution was added to the reaction system to initiate the reaction, and the rate of absorbance decrease per minute was recorded. The enzyme activity is determined by the decrease in absorbance at 290 nm per minute (Nakano and Asada, 1981).

#### 2.3.6 Determination of yield-related indicators

At the time of the rice harvest, 20 randomly selected representative plants, free of pests and diseases, were selected from each treatment. The panicle length, panicle number per hill, and filled grain number were measured, and the seed setting rate was calculated. The 1000-grain weight and yield per plant of rice were examined after air-drying.

### 2.4 Statistical analysis

Variance analysis was performed on the data for each sample using the Excel (2016, Microsoft Corp., Redmond, WA, USA) and SPSS (26.0, IBM Corp., Armonk, NY, USA). The mean value was tested by the least significant difference at the P< 0.05 level (LSD 0.05). All data are expressed as mean ± standard deviation (SE) of multiple replicates. Origin 2021 was used for drawing.

## 3 Result

### 3.1 Effects of exogenous Uniconazole on shoot growth of rice seedlings under salt stress

**Figure 1:**
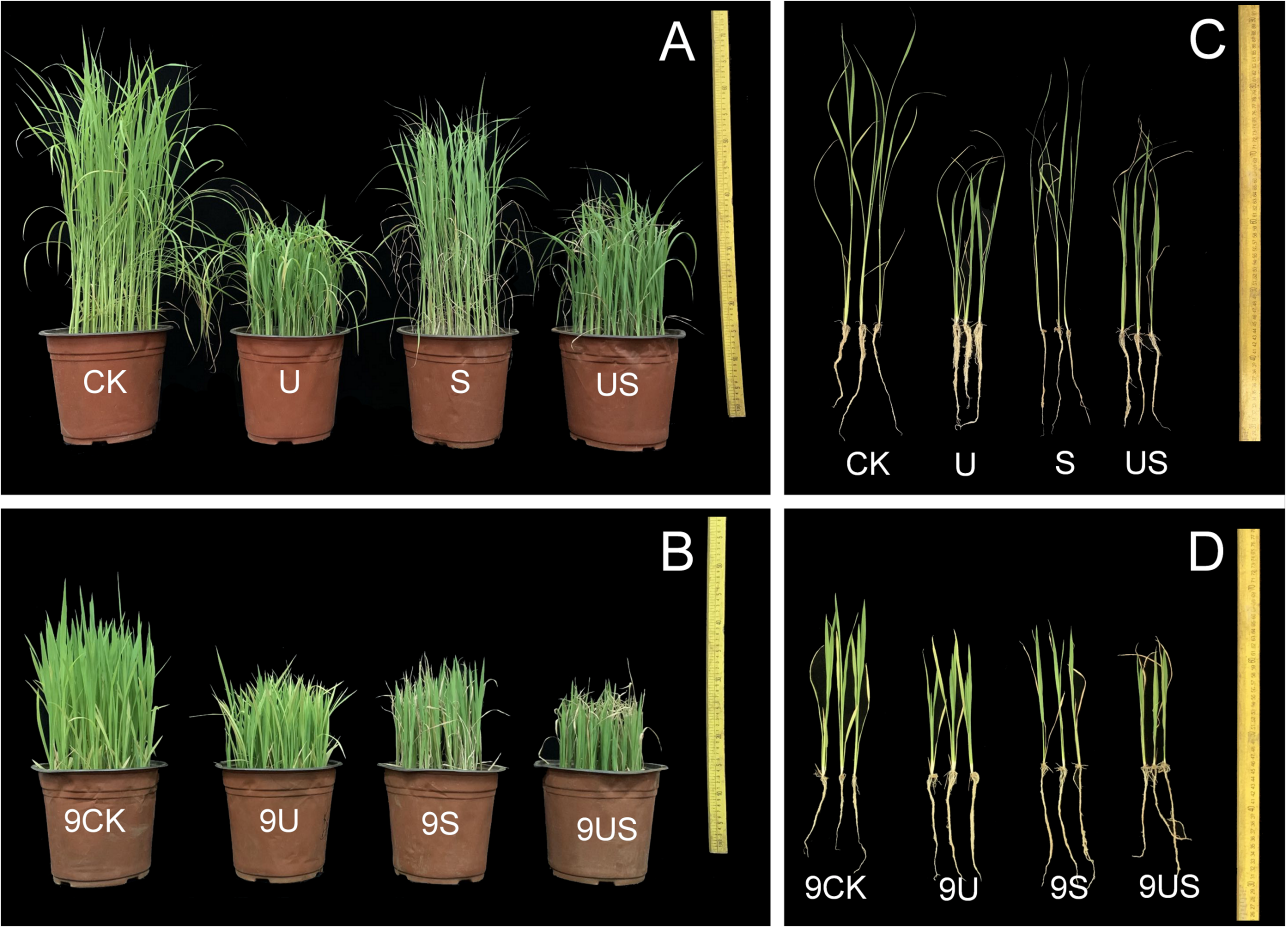
Effects of exogenous uniconazole on the growth of rice seedlings HD961 and 9311 under salt stress. **(A)** and **(B)** depict a repeated whole growth diagram (a plastic pot) for each treatment of HD961 and 9311, while **(C)** and **(D)** show three seedling growth morphology diagrams for each treatment of HD961 and 9311. The experimental treatments were as follows: (1) CK, 0% NaCl + Spraying water; (2) U, 0% NaCl + Spraying 10 mg·L^-1^ Uniconazole; (3) S, 0.6% NaCl + Spraying water; (4) US, 0.6% NaCl + Spraying 10 mg·L^-1^ Uniconazole.

**Figure 2:**
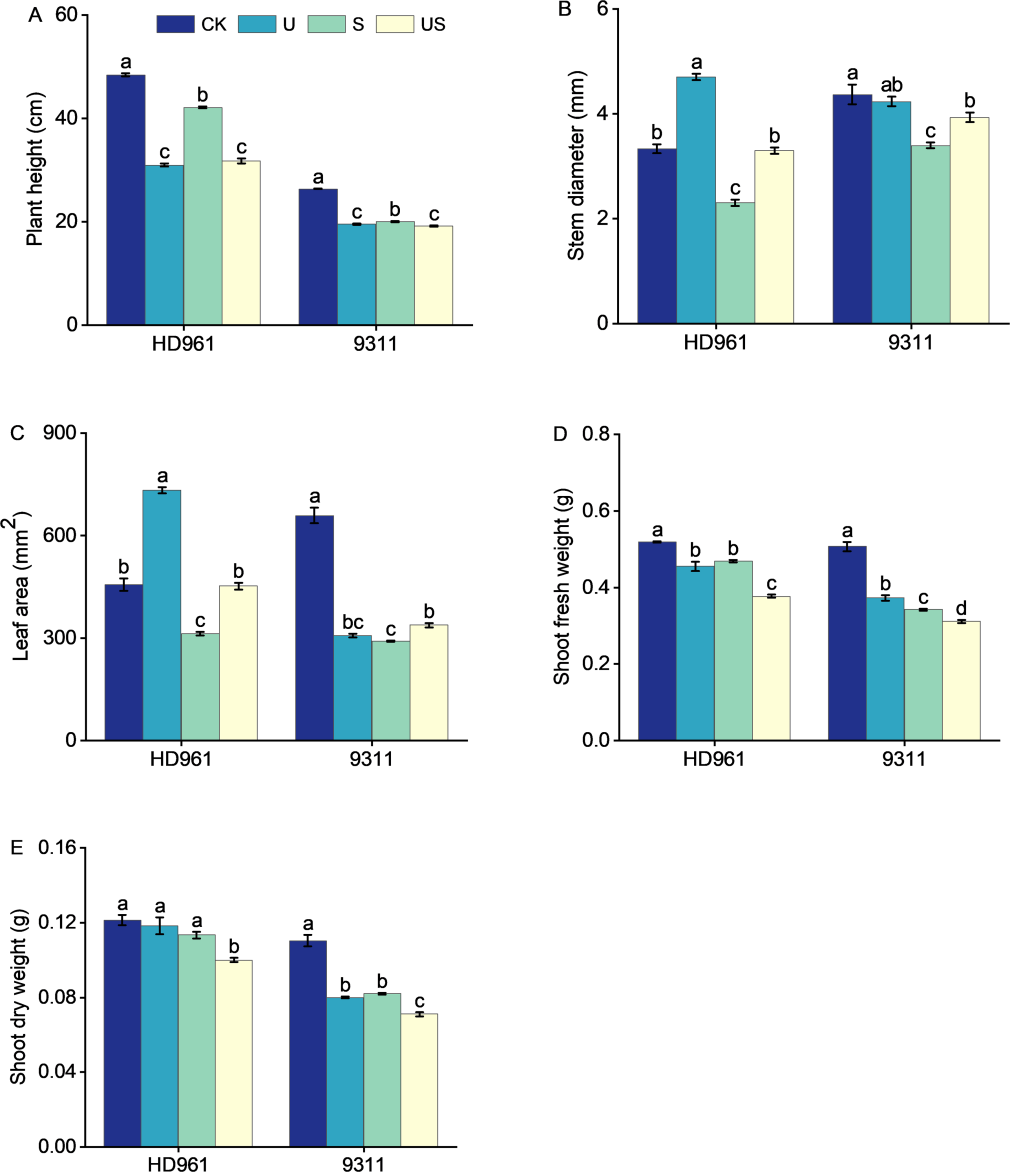
Effects of exogenous uniconazole on shoot growth of rice seedlings HD961 and 9311 under salt stress. Plant height of HD961 and 9311 **(A)**, stem diameter of HD961 and 9311 **(B)**, leaf area of HD961 and 9311 **(C)**, shoot fresh weight of HD961 and 9311 **(D)**, shoot dry weight of HD961 and 9311 **(E)**. The value represents the average ± standard error (SE) of the three repeated samples. According to the Duncan test, different letters in the data column indicate significant differences (p< 0.05).

Rice growth was strongly suppressed under salt stress (**Figure 1**). The stem diameter, leaf area, shoot dry weight, and fresh weight of the two varieties were all decreased significantly (**Figure 2**). The application of exogenous uniconazole alleviated this damage. There were corresponding percent decreases of 13.0% and 24.2% in plant height, 31.0% and 22.1% in stem diameter, 31.6% and 55.8% in leaf area, 9.8% and 32.5% in fresh weight of shoot, 6.6% and 25.5% in fresh weight of shoot, respectively for HD961 and 9311. The plant height, stem diameter, leaf area, fresh weight of shoot, and dry weight of shoot of both varieties are significantly affected by Uniconazole. Compared with salt stress, US treatment decreased the percentage to 24.5% and 4.0% in plant height, 19.4% and 8.8% in fresh weight of shoot, and 11.5% and 13.4% in dry weight of shoot, respectively for HD961 and 9311. The stem diameter and leaf area of both varieties are significantly increased by Uniconazole under salt stress. There were corresponding percent increases of 43.5% and 15.7% in stem diameter, 44.7% and 16.2% in leaf area, respectively for HD961 and 9311. The data showed that the inhibitory effect of salt stress on the aboveground section of 9311 was greater than on HD961.

### 3.2 Effects of exogenous Uniconazole on root growth of rice seedlings under salt stress

**Figure 3:**
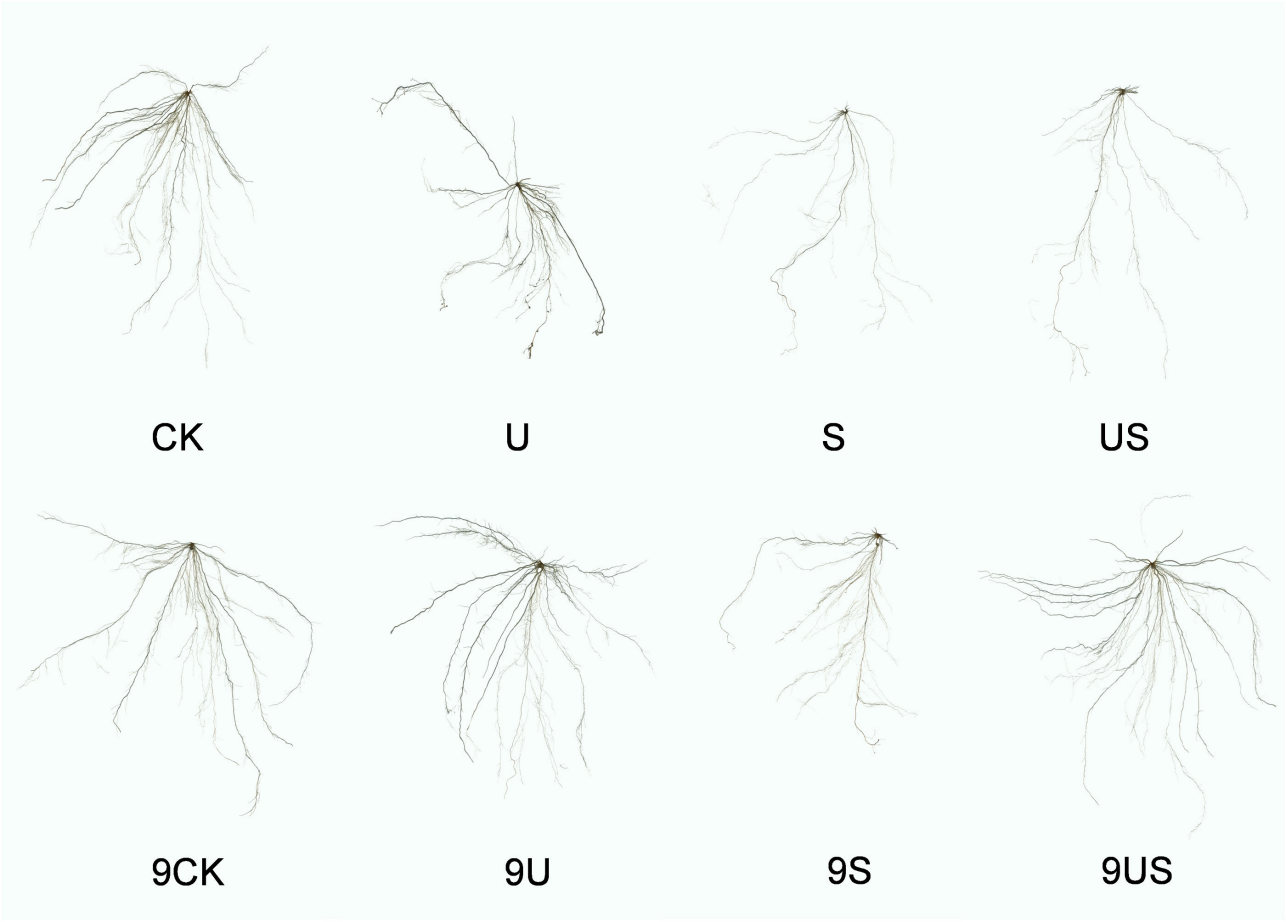
Root morphology of exogenous uniconazole rice seedlings under salt stress. The experimental treatments were as follows: (1) CK, 0% NaCl + Spraying water; (2) U, 0% NaCl + Spraying 10 mg·L^-1^ Uniconazole; (3) S, 0.6% NaCl + Spraying water; (4) US, 0.6% NaCl + Spraying 10 mg·L^-1^ Uniconazole. Among them, CK, U, S, and US represent the rice variety HD961, while 9CK, 9U, 9S, and 9US represent the 9311 rice varieties.

**Figure 4:**
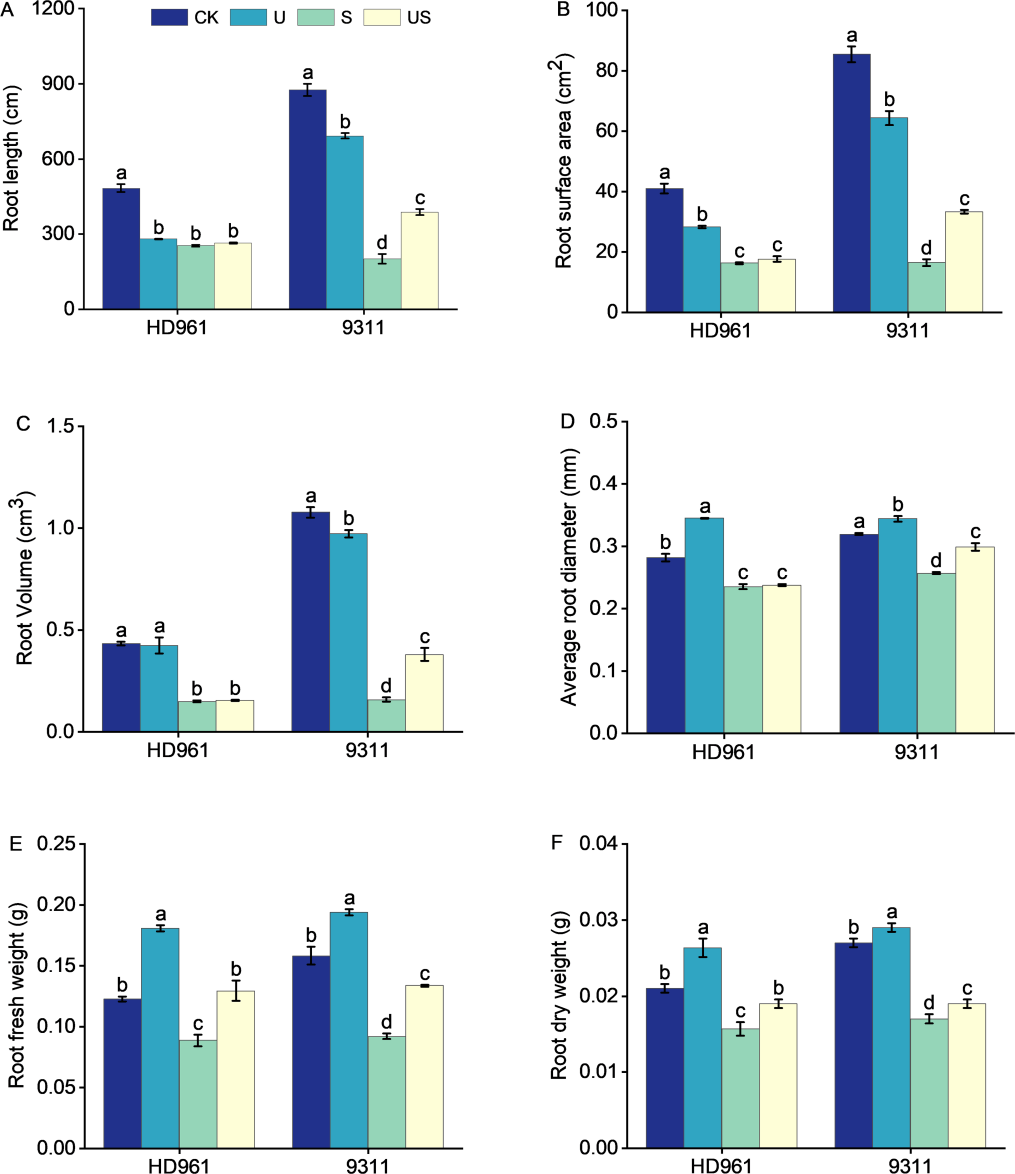
Effects of exogenous uniconazole on root formation of rice seedlings HD961 and 9311 under salt stress. Root length of HD961 and 9311 **(A)**, root surface area of HD961 and 9311 **(B)**, root volume of HD961 and 9311 **(C)**, average root diameter of HD961 and 9311 **(D)**, root fresh weight of HD961 and 9311 **(E)**, root dry weight of HD961 and 9311 **(F)**. The value represents the average ± standard error (SE) of the three repeated samples. According to the Duncan test, different letters in the data column indicate significant differences (p< 0.05).

**Figure 3** indicates that the root growth of the two rice varieties was greatly inhibited under salt stress, which was significantly improve following the exogenous application of Uniconazole. Compared to CK, salt stress significantly decreased the percentage to 47.5% and 77.1% in root length, 60.2% and 80.7% in root surface area, 65.7% and 85.3% in root volume, 16.3% and 19.4% in root average diameter, 27.6% and 41.8% in root fresh weight, and 23.8% and 37.0% in root surface area, respectively, for HD961 and 9311. As can be seen, salt stress had a greater inhibitory effect on the roots of 9311 than on HD961. Compared to S treatment, US treatment significantly increased the percentage to 93.2% in root length, 102.1% in root surface, 139.0% root volume, 16.3% in root average diameter, 45.7% in root fresh weight, and 11.8% in root dry weight of variety 9311. Compared to S treatment, US treatment significantly increased the root fresh weight (44.9%) and root dry weight (18.8%) of variety HD961. There were corresponding percent increases of 3.9% in root length, 7.8% in root surface area, 4.7% in root volume, and 0.8% in root average diameter of HD961 (**Figure 4**). It can be seen that Uniconazole was having strongly regulatory effect on 9311 thanHD961 under salt stress.

### 3.3 Effects of exogenous Uniconazole on chlorophyll content of rice seedlings under salt stress

**Figure 5:**
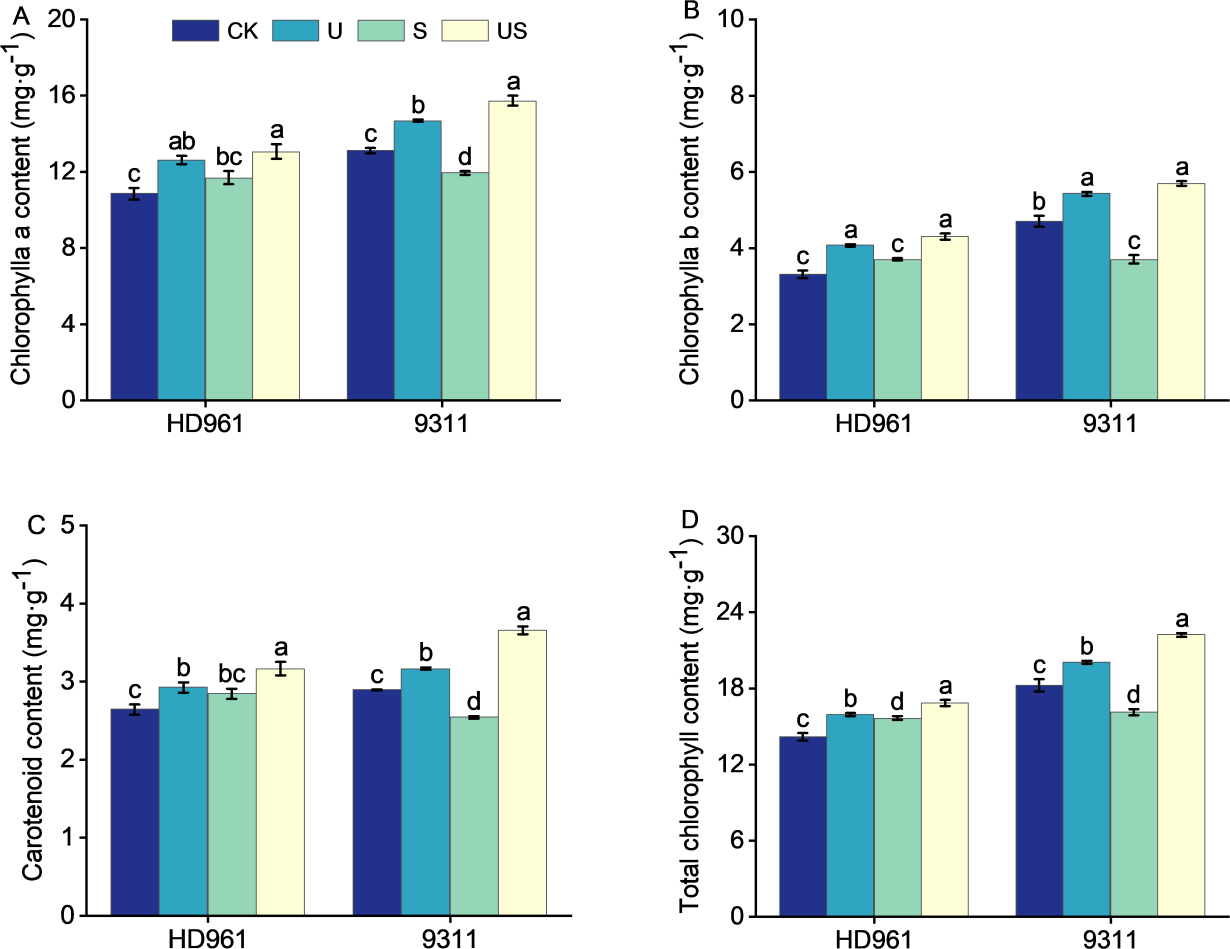
Effects of exogenous uniconazole on chlorophyll content of rice seedlings under salt stress. Chlorophyll a content in HD961 and 9311 **(A)**, chlorophyll b content in HD961 and 9311 **(B)**, carotenoid content in HD961 and 9311 **(C)**, and total chlorophyll content in HD961 and 9311 **(D)**. The value represents the average ± standard error (SE) of the three repeated samples. According to the Duncan test, different letters in the data column indicate significant differences (p< 0.05).

Compared to CK, salt stress increased the chlorophyll a content by 7.8%, chlorophyll b content by 12.0%, carotenoids content by 7.7%, and total chlorophyll content by 10.3%, respectively for HD961 (**Figure 5**). In 9311, however, the trend is in the opposite direction. Adversely, compared to CK, salt stress markedly decreased the chlorophyll a content by 8.9%, chlorophyll b content by 21.2%, carotenoids content by 12.1%, and total chlorophyll content by 11.7, respectively for 9311. US treatment increased the chlorophyll a content and carotenoids content, and significantly increased the chlorophyll b content (16.0%) and total chlorophyll content (7.5%), respectively, for HD961, compared to salt stress. Similarly, compared with S, US treatment significantly increased the chlorophyll a content by 31.7%, chlorophyll b content by 53.9%, carotenoids content by 43.7%, and total chlorophyll content by 38.0%, respectively for 9311 It is clear that the effect of exogenously spraying Uniconazole on chlorophyll content in 9311 leaves was superior to that of HD961 during salt stress.

### 3.4 Effects of exogenous Uniconazole on the activities of antioxidant enzyme system related enzymes in rice under salt stress

**Figure 6:**
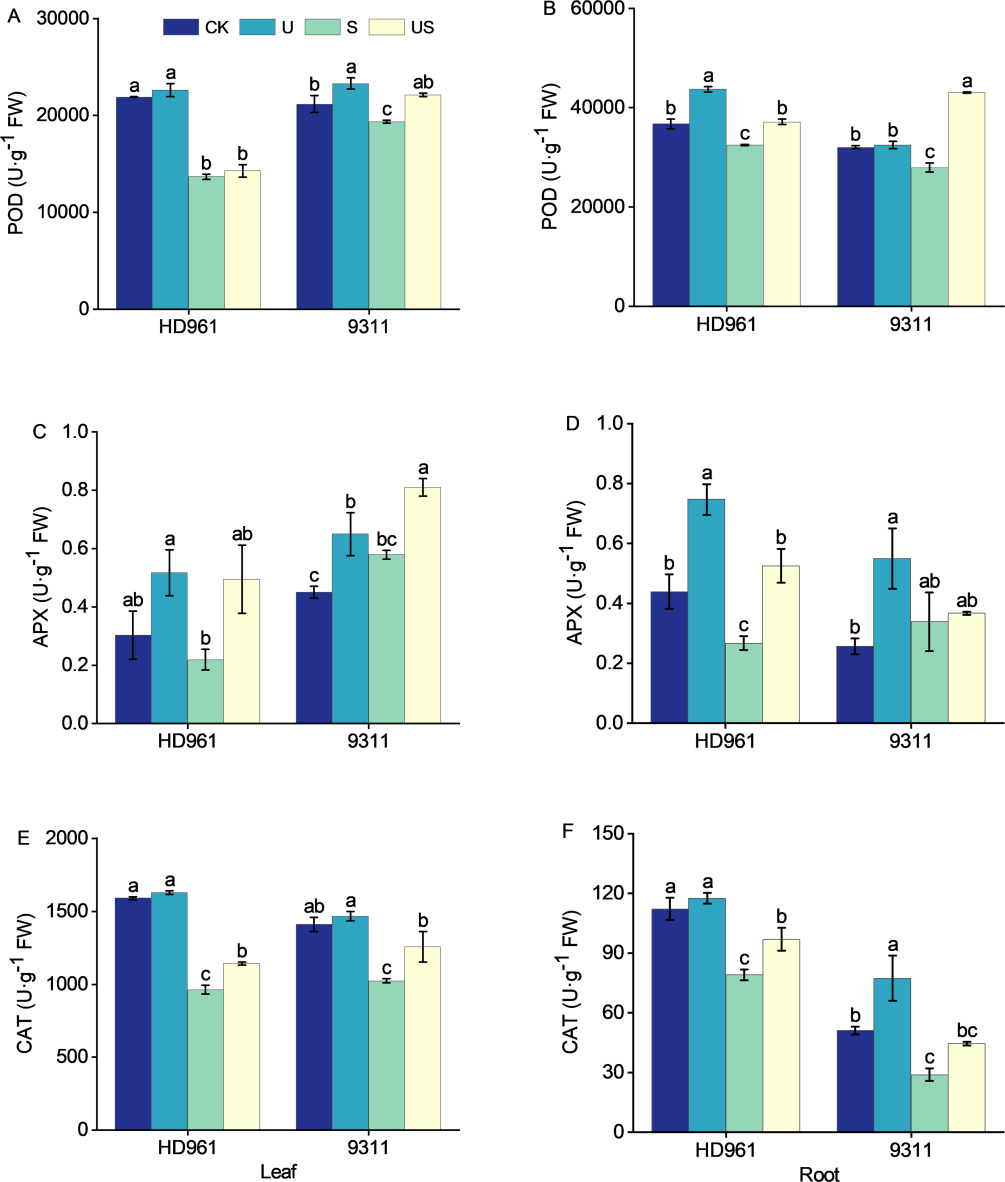
Effects of exogenous uniconazole on antioxidant enzyme activities in rice under salt stress. The activities of the POD enzyme in leaves **(A)** and roots **(B)** for HD961 and 9311, the APX enzyme activities in leaves **(C)** and roots **(D)** for HD961 and 9311, and the CAT enzyme activities in leaves **(E)** and roots **(F)** for HD961 and 9311. The value represents the average ± standard error (SE) of the three repeated samples. According to the Duncan test, different letters in the data column indicate significant differences (p< 0.05).

**Figure 6** depict the activity of antioxidant enzymes (POD, APX, and CAT) in rice leaves and roots following various treatments. Compared with CK, salt stress decreased the activities of POD and CAT in the leaves and roots of both cultivars (**Figures 6A, B, E, F**). It decreased the APX activity in the leaves and roots of HD961, while increasing the APX activity in the leaves and roots of 9311 (**Figures 6C, D**). Compared to salt stress US treatment increased the percentage by 4.5% and 14.3% in POD activity of leaves, by 126.8% and 39.8% in APX activity of leaves, by 18.7% and 22.7% in CAT activity of leaves, by 14.4% and 54.2% in POD activity of roots, by 96.6% and 8.3% in APX activity of roots, and by 22.6% and 53.9% in CAT activity of roots, respectively, for HD961 and 9311. Overall, spraying Uniconazole under salt stress modulated the enzyme activities of roots more effectively than those of leaves in both varieties.

### 3.5 Effects of exogenous Uniconazole on photosynthesis of rice HD961 at the booting stage and the full heading stage under salt stress

**Figure 7:**
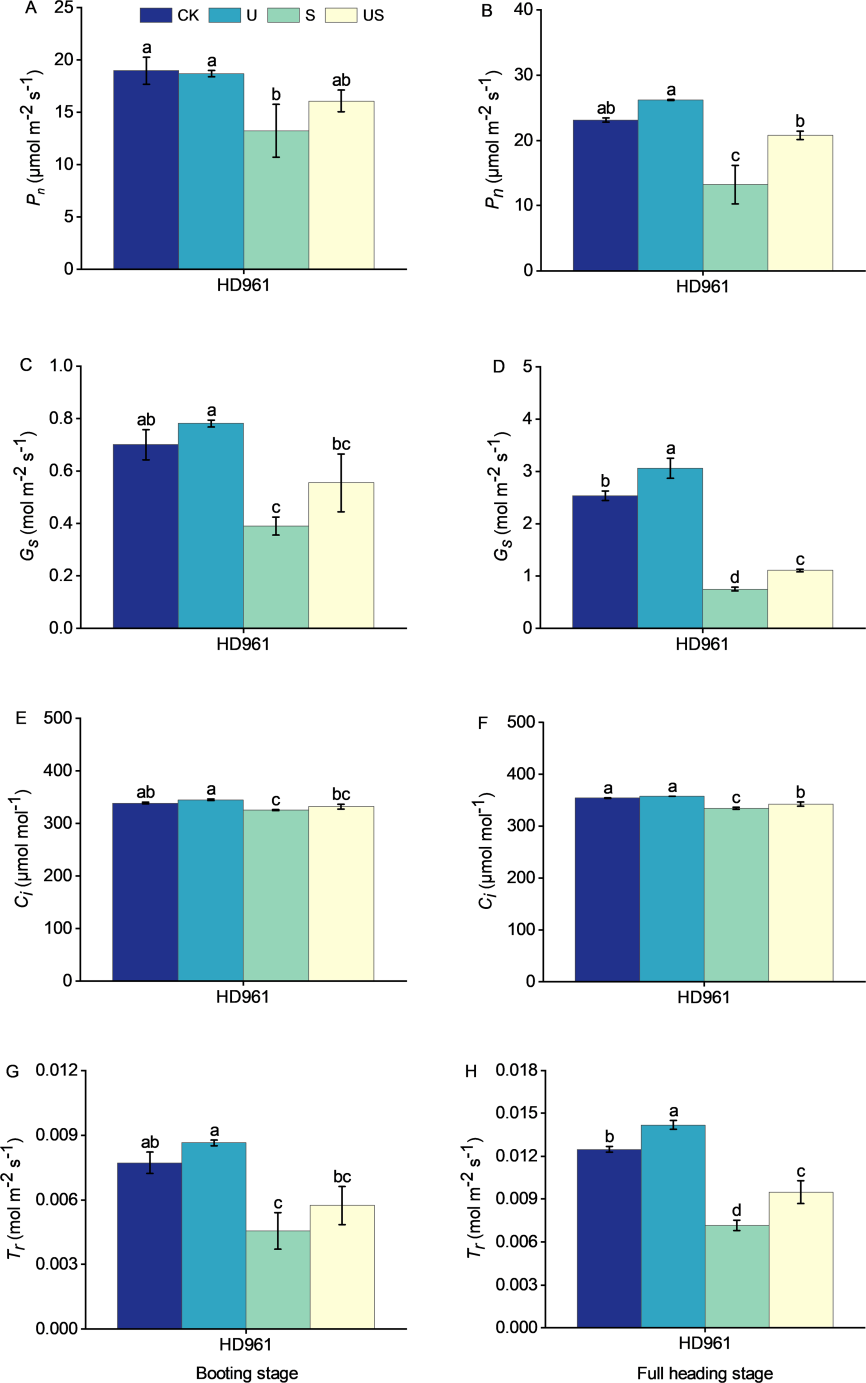
Effects of exogenous uniconazole on the photosynthesis of rice variety HD961 during the booting stage and full heading stage under salt stress. The net photosynthetic rate of HD961 at the booting stage **(A)** and full heading stage **(B)**, stomatal conductance of HD961 at the booting stage **(C)** and full heading stage **(D)**, intercellular carbon dioxide concentration of HD961 at the booting stage **(E)** and full heading stage **(F)**, and transpiration rate of HD961 at the booting stage **(G)** and full heading stage **(H)**. The value represents the average ± standard error (SE) of the three repeated samples. According to the Duncan test, different letters in the data column indicate significant differences (p< 0.05).

The effects of salt and Uniconazole treatment on photosynthesis at the booting stage and full heading stage are shown in **Figure 7**. Salt stress significantly reduced the photosynthesis of HD961. There were corresponding percentages of 30.3% and 42.7% in *P_n_*, 44.3% and 70.4% in *G_s_*, 4.0% and 5.5% in *C_i_*, and 41.1% and 42.7% in *T_r_*, respectively, in the booting stage and full heading stage for HD961. Uniconazole application under salt stress boosted photosynthesis at both the booting and full heading stages, with the full heading stage showing the most significant increase. Compared to S treatment, the US treatment increased the percentage by 21.5% and 57.3% in *P_n_*, by 42.4% and 47.5% in *G_s_*, by 2.1% and 2.4% in *C_i_*, and by 26.1% and 32.8% in *T_r_*, respectively, in the booting stage and full heading stage for HD961. This shows that Uniconazole could enhance photosynthesis, promote grain filling, and mitigate the adverse effects of salt stress on rice growth and yield.

### 3.6 Effects of exogenous Uniconazole on the yield of rice HD961 under salt stress

**Figure 8:**
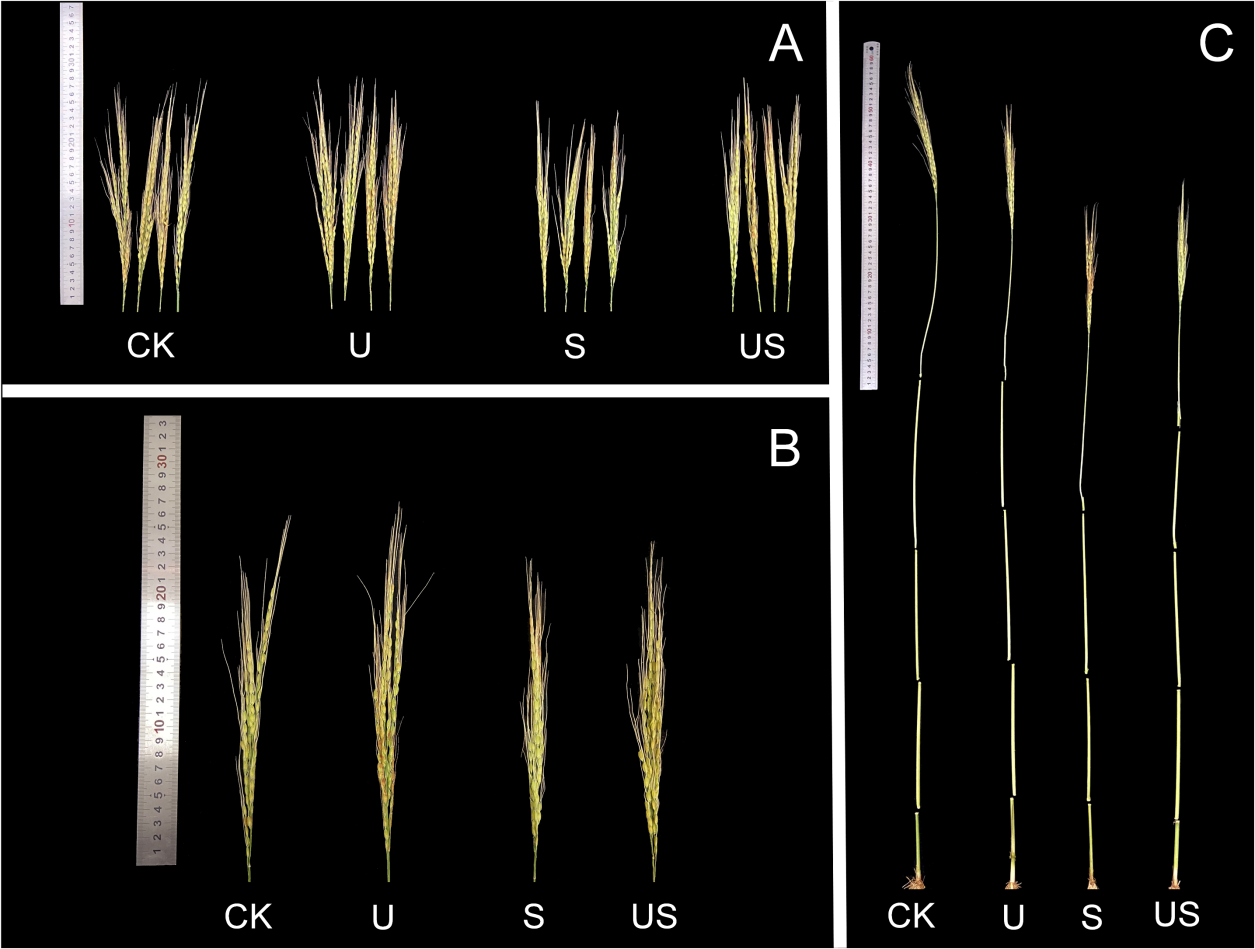
Effects of exogenous Uniconazole on the full heading stage of rice HD961 under salt stress. **(A)** and **(B)** are the panicles of rice HD961, while **(C)** represents the aerial part of the HD961 plant. The photograph was taken on January 8, 2023, at the College of Coastal Agriculture, Guangdong Ocean University.

**Figure 9:**
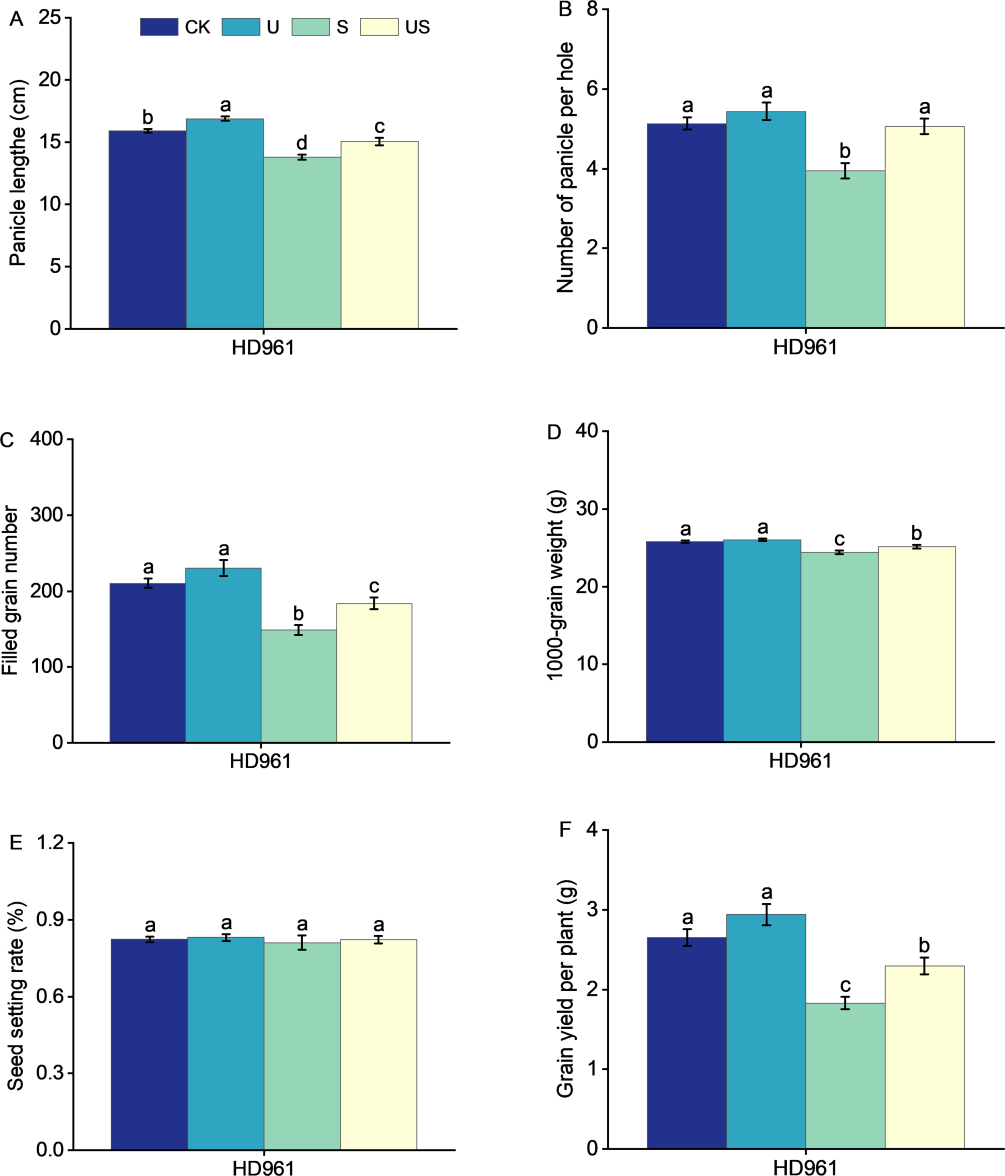
Effects of exogenous Uniconazole on the full heading stage of rice HD961 under salt stress. Panicle length **(A)**, panicle number per hole **(B)**, filled grain number **(C)**, 1000-grain weight **(D)**, seed setting rate **(E)**, and yield per plant **(F)** of rice variety HD961. The value represents the average ± standard error (SE) of the sixteen repeated samples. According to the Duncan test, different letters in the data column indicate significant differences (p< 0.05).

Compared to the S treatment, the U treatment significantly shortened the internode length and promoted the panicle growth of HD961 (**Figure 8**). **Figure 9** shows that salt stress significantly reduced the height of rice plants and the development of panicles. There were corresponding percent decreases of 13.0% in panicle length (PL), 23.0% in panicle number per hole (PNH), 29.0% in filled grain number (FGN), 5.0% in 1000-grain weight (1000-GW), and 31.0% in yield per plant (YP), respectively, for HD961. Compared to salt stress, Uniconazole + salt stress treatment significantly increased the percentage to 9.0% in PL, 28.0% in PNH, 24.0% in FGN, 3.0% in 1000-GW, and 26.0% in YP, respectively, for HD961. While there was no significant increase in the seed setting rate for HD961.

### 3.7 Correlations between growth and physiological parameters and rice yield in HD961

**Figure 10:**
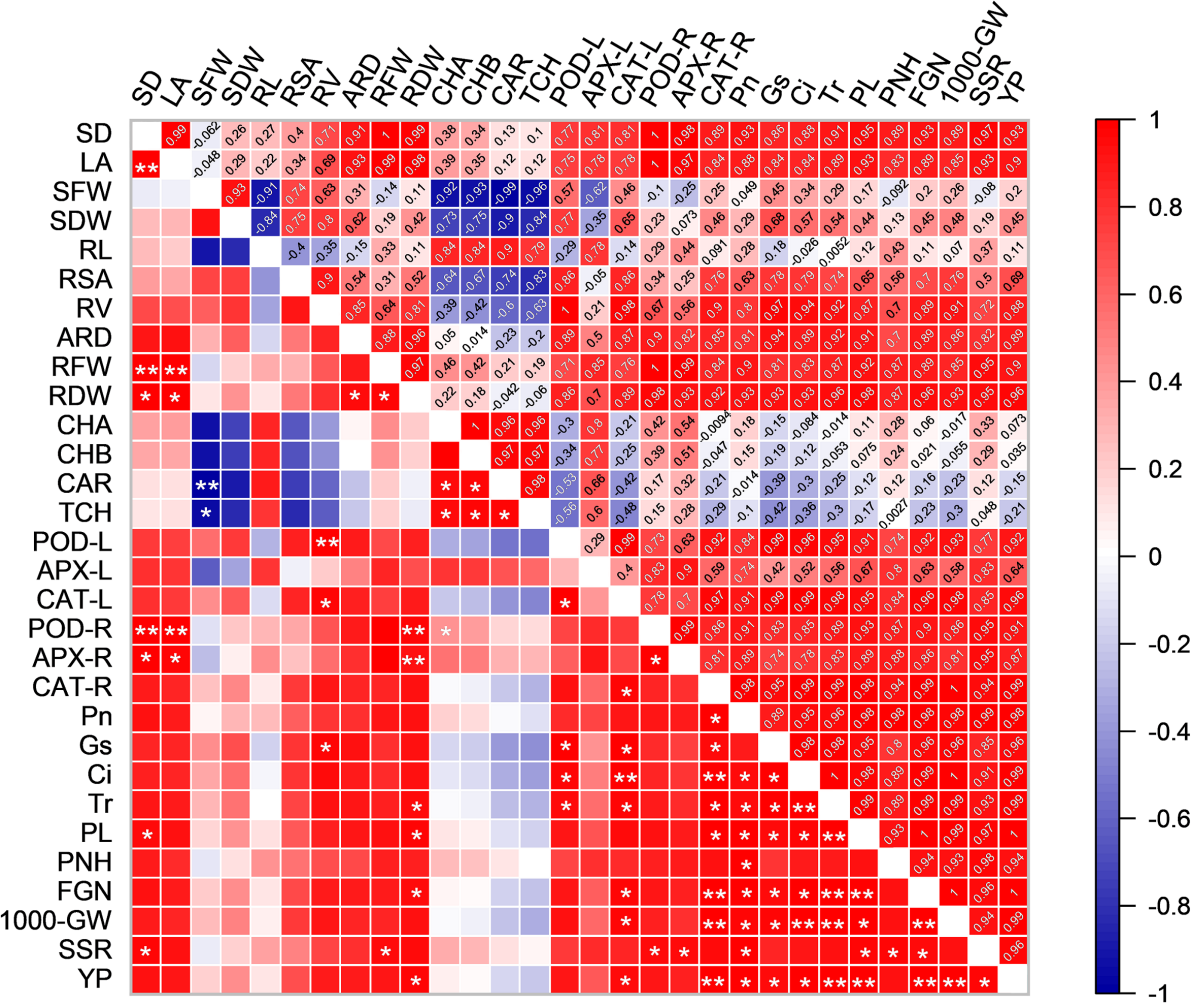
Pearson correlations between growth and physiological parameters and rice yield in HD961. Red indicates a positive correlation between the two parameters. Blue indicates a negative correlation between the two parameters. SD, LA, SFW, SDW, RL, RSA, RV, ARD, RFW, RDW, CHA, CHB, CAR, TCH, POD-L, APX-L, CAT-L, POD-R, APX-R, CAT-R, *P_n_*, *G_s_*, *C_i_*, *T_r_*, PL, PNH, FGN, 1000-GW, SSR and YP represent stem diameter, leaf area, shoot fresh weight, shoot dry weight, root length, root surface area, root volume, average root diameter, root fresh weight, root dry weight, chlorophyll a, chlorophyll b, carotenoid, total chlorophyll, POD activity in leaves, APX activity in leaves, CAT activity in leaves, POD activity in roots, APX activity in roots, CAT activity in roots, *P_n_*, *G_s_*, *C_i_*, *T_r_*, panicle length, panicle number per hole, filled grain number, 1000-grain weight, seed setting rate and yield per plant, respectively. ANOVA p values and symbols were defined as: * p< 0.05; ** p< 0.01; ns: p > 0.05.

To better understand the relationship between growth and physiological parameters and yield-related traits, we created a correlation matrix and compared the correlations between the indicators (**Figure 10**). The PL was positively correlation with RDW, POD-L, CAT-L, CAT-R, *P_n_*, *G_s_*, *C_i_* and *T_r_*. The YP was positively correlation with RDW, POD-L, CAT-L, CAT-R, *P_n_*, *G_s_*, *C_i_*, *T_r_*, PL, 100-GW and SSR. Similarly, PL, FGN, 1000-GW, SSR and YP are all positively correlation with RDW, POD-L, CAT-L, CAT-R, *P_n_*, *G_s_*, *C_i_* and *T_r_*. This indicates a positive correlation between rice yield, photosynthesis, and antioxidant enzyme activities.

## 4 Discussion

Excessive soil salinity interferes with the normal physiological, biochemical, and metabolic processes of the plant, which adversely affects its growth and development (Talubaghi et al., 2023). Above ground is an essential metabolic and synthesizing organ of plants, the growth status of the above-ground part can reflect the salt tolerance of plants. The better the growth status of above ground, the stronger the salt tolerance of plants. Many studies have shown that plant leaf and root growth is inhibited in high-salt environments (**Figure 1**), which ultimately reduces biomass accumulation (Shahid et al., 2020). This study confirmed that salt stress severely inhibited the growth and development of rice, leading to a reduction in the shoot fresh weight and dry weight of HD961 and 9311. Notably, the salt-sensitive rice variety 9311 declined more than the salt-tolerant rice variety HD961, indicating that the salt-sensitive variety 9311 is more susceptible to salt stress injury. The inhibition and disruption of photosynthesis may be the primary cause of the decline in rice biomass during salt stress (**Figure 7**). The accumulation of Na and K ions within the cell, which obstruct relevant synthesis and metabolic processes (Gerona et al., 2019; Yang et al., 2019), could also be the cause. While foliar application of Uniconazole reduced the plant height, shoot fresh weight, and dry weight of HD961 and 9311, which was consistent with the previously reported effects of Uniconazole on rice (Izumi et al., 1984). Studies have shown that uniconazole can effectively increase stem diameter and reduce plant height to protect plants from damage (Fletcher et al., 1999; Schluttenhofer et al., 2011). The results of this study show that the US treatment significantly reduced the plant height, while significantly increasing the stem diameter and leaf area of the two rice varieties, compared to salt stress (**Figure 2**). These findings are consistent with the results reported by Hussein and Bekheta et al (Hussein et al., 2015). Moreover, the application of Uniconazole under salt stress reduced the plant height of HD961 more than that of 9311, which indicated that spraying Uniconazole under salt stress could effectively reduce the plant height of taller plants, enhance lodging resistance (Meichen et al., 2023), and improve the yield to a certain extent. It was reported that the application of Uniconazole could alleviate the damage of salt stress on plants by increasing the root fresh and dry weights of plants under salt stress, which is consistent with our findings (Ningyi et al., 2011). It was shown that Uniconazole could alleviate the damage caused by salt stress by reducing the shoot height of rice, increasing stem diameter and leaf area, and achieving the seedling strengthening effect under salt stress.

The root system is an important organ for plants to absorb nutrients and water from the soil, while its growth and development is sensitive to salt stress (Gorham et al., 1985). Therefore, the growth status of roots can directly indicate the damage caused by soil salt stress to plants. The results of this study showed that salt stress significantly reduced the root length, root surface area, root volume, average root diameter, and the root fresh weight and dry weight of rice varieties HD961 and 9311 (**Figure 4**). This indicates that salt stress inhibited rice root growth, and this result is consistent with the research findings of Mu et al. (Mu et al., 2022) on rice. It is worth noting that the reduction in root growth of 9311 than in HD961, which is consistent with the findings of previous study on rice, which found that salt-sensitive phenotypes inhibited rice root morphology (total root length, root surface area, root volume, and average root diameter) more effectively (Mu et al., 2022). This indicates that salt-sensitive rice varieties are more susceptible to damage caused by salt stress than salt-tolerant rice varieties. Compared to salt stress, US treatment significantly increased the root growth-related indexes of HD961 and 9311, which was similar to the findings of Qiu et al.’s (Qiu et al., 2005) study on promoting root growth in rapeseed under waterlogging stress using Uniconazole. Another study reported that foliar spraying of Uniconazole could also improve salt tolerance by effectively altering the number and caliber of secondary xylem conduits in soybean roots under salt stress, thereby reducing the entry of harmful ions into the roots (Na et al., 2017). In addition, this paper showed that foliar spraying of Uniconazole was more effective in promoting the root growth condition of salt-sensitive rice 9311 under salt stress, whereas previous studies also showed that application of the plant growth regulator BR under salt stress was better than salt-tolerant rice in regulating salt-sensitive rice (Mu et al., 2022). Analyzing the effects of Uniconazole on the shoot and root morphology of two rice varieties under salt stress demonstrates that Uniconazole can regulate shoot components and promote root development (Yan et al., 2013), thereby alleviating the damage caused by salt stress.

The leaf is a vital organ for photosynthesis and the formation of organic matter, and the chlorophyll content in leaves can reflect the photosynthetic capacity of plants in a certain extent (Wang et al., 2017). Maintaining a stable chlorophyll content enhances photosynthesis and increases plant resistance to stress (Kostopoulou et al., 2015). The results of this study showed that salt stress increased the chlorophyll a, chlorophyll b, carotenoid and total chlorophyll content of HD961 (**Figure 5**). This could be because salt stress inhibits the growth of shoots for HD961, resulting in a lower plant height than CK. This results in a decrease in the dispersion range of pigments while increasing chlorophyll content. Adversely, salt stress reduced the levels of chlorophyll a, chlorophyll b, carotenoids, and total chlorophyll content in rice variety 9311. The decrease in chlorophyll content in 9311 could be attributed to increased activity of the Chlorophylls enzyme and the instability of the pigment-protein complex (Beinsan et al., 2009). This shows that a salt-stress environment may destroy the chloroplast structure of rice leaves, impede the synthesis of chlorophyll, or accelerate the degradation of pigments (Qu et al., 2012; Hameed et al., 2021). Furthermore, previous research have also shown that salt-tolerant rice genotypes are superior to salt-sensitive genotypes in retaining chlorophyll content (Cha-Um et al., 2009). Compared to salt stress, the US treatment increased the contents of chlorophyll a, chlorophyll b, carotenoids, and total chlorophyll content in HD961 and 9311, respectively, which was consistent with the effects of Uniconazole application on maize in saline-alkali land (Xu et al., 2022). It is possible that under stress conditions, the zeatin content and chloroplast size of plants treated with Uniconazole rose, and plants were greener due to the higher chlorophyll content (Fletcher and Arnold, 2010; Gao et al., 1988). Similarly, previous studies reported that the application of Uniconazole under salt stress can prevent ROS damage to chloroplasts and significantly inhibit chlorophyll catabolism, thereby increasing plant chlorophyll content (Caihong, 2022). In addition, leaf spraying of Uniconazole under stress increased cytokinin (CK) biosynthesis, up-regulated the expression of key enzymes in the chlorophyll biosynthesis pathway, and reduced the degradation of photosynthetic pigments, all of which increased chlorophyll content (Liu et al., 2015). In this regard, studies have demonstrated that foliar spraying of Uniconazole under cold stress delays the degradation of chlorophyll in rape, enhances the respiratory capacity of the root system, and boosts plant resistance (Zhou and Leul, 1998). The research results (**Figure 5**) show that the salt-sensitive variety 9311 has higher chlorophyll content than the salt-tolerant variety HD961. We speculate that this result is due to Uniconazole’s variable effects on the leaf senescence process in rice varieties with different salt tolerance under salt stress. It could also be attributed to Uniconazole’s variable ability to degrade chlorophyll enzymes, which affects the level of chlorophyll and carotenoids differentially in different rice cultivars.

Photosynthesis is essential for plant growth and yield (Yang et al., 2022), and 90% of rice grain yield comes from the accumulation of photosynthetic products in functional leaves after flowering (Roháek and Barták, 1999). Therefore, maintaining photosynthesis is one of the important mechanisms for plants to adapt to salt tolerance (Fan et al., 2007). Leaf senescence is a widespread natural phenomenon. However, saline-alkali stress can severely affect the pace and timing of leaf senescence (Allu et al., 2014). In addition, salt stress further increases the toxicity of rice leaves, leading to leaf senescence and reduced leaf area, which ultimately reduces the photosynthetic rate (Hussain et al., 2018; Munns, 2002). In this study, salt stress significantly reduced the photosynthetic parameters such as *P_n_*, *G_s_*, *C_i_*, and *T_r_* of rice variety HD961 at the booting stage and full heading stage (**Figure 7**), indicating that salt stress had an effect on photosynthesis and stomatal closure of plants. At the same time, the overall photosynthetic rate of rice was reduced due to salt stress reducing photosynthetic pigments (chlorophyll a, b, and carotenoid content) in the plant (Acosta-Motos et al., 2017). This is due to the fact that salt stress causes physiological drought in plants. Plants are unable to maintain normal photosynthesis due to reduced CO_2_ uptake in order to minimize water loss from the body (Suzuki et al., 2012). The decrease in *C_i_* in rice under salt stress was accompanied by a simultaneous decrease of *P_n_* and *G_s_* (**Figure 7**), which indicated that salt stress caused the decrease of *P_n_* in rice through stomatal limitation (Tang et al., 2018; Yang et al., 2006). In addition, salt-stressed environments may destroy the chloroplast structure of rice leaves, impede chlorophyll synthesis, or accelerate pigment degradation, ultimately leading to reduced photosynthetic activity (Qu et al., 2012). It has been reported that when plants are subjected to salt stress, the chloroplast membrane system is destroyed to a certain extent, leading to chloroplast decomposition, which eventually results in a decrease in the net photosynthetic rate (Gadelha et al., 2021). Under salt stress, exogenously spraying Uniconazole increased the photosynthetic parameters of rice at the booting stage and full heading stage, enhanced *P_n_*, and maintained photosynthesis. This is consistent with the results of previous studies in which Uniconazole was applied to enhance the photosynthetic properties of plants under water stress (Ningyi et al., 2012). In addition, as shown in **Figure 7**, the improvement effect of US treatment on the photosynthetic parameters of HD961 at the full heading stage was better than at the booting stage when compared to salt stress. This may be due to the role of Uniconazole in delaying leaf senescence and further enhancing photosynthesis in rice at the full heading stage (Wan et al., 2013), promoting more efficient transport of photosynthetic products to the grains, and ultimately increasing yield. This may also be because Uniconazole induced stomatal opening, which affects the diffusion of CO_2_ from the external environment through the stomatal opening to the mesophyll tissue, thereby increasing the rate of photosynthetic carbon assimilation. Meanwhile, correlation analysis showed that photosynthesis and chlorophyll content were positively correlated (**Figure 10**), whereas spraying Uniconazole under salt stress increased chlorophyll content in rice leaves (**Figure 5**), which improved photosynthetic efficiency to some extent. The above results indicate that Uniconazole was able to maintain the chloroplast structure and enhance the photosynthetic capacity of rice, thereby alleviating the inhibitory impact of salt stress on rice.

Abiotic stresses, such as salt stress, can damage the oxygen-evolving complex (OEC) in the process of electron transfer in plants. This damage leads to the generation of large amounts of reactive oxygen species (ROS) in plants, destroying macromolecules such as proteins and nucleic acids, and causing membrane lipid peroxidation, destroying the normal physiological metabolism process in plants and even inducing programmed cell death (Miller et al., 2010). There is an efficient antioxidant defense system in plants for balancing and scavenging excess reactive oxygen species (ROS) produced under stress, including antioxidant enzymes such as POD, APX, and CAT (Alam et al., 2019). Moreover, H_2_O_2_ is decomposed by POD, APX, and CAT to form molecular oxygen and water, which prevents membrane lipid peroxidation, delays plant senescence, and maintains normal growth and development. Studies have shown that triazoles can reduce membrane oxidative damage and improve plant tolerance to stress by increasing antioxidant activity (Fletcher et al., 1999). This study showed that the activities of POD and CAT in the leaves and roots of HD961 and 9311, were significantly reduced by salt stress. This reduction may be attributed to the rapid overproduction of ROS under high salt stress, which exacerbates membrane lipid peroxidation, destroys the stability of the cell membrane, causes damage to the antioxidant defense system, and ultimately leads to plant damage under salt stress. This is consistent with previous research findings (Mu et al., 2022). In this study, we observed higher antioxidant enzyme activities in 9311 leaves compared to HD961 under salt stress. We hypothesized that this difference might be due to the fact that the negative impacts of salt stress on the growth of salt-sensitive rice varieties were not primarily caused by salt-induced oxidative stress (Vicent et al., 2003). This study showed that the enzyme activities of POD, APX, and CAT increased in the leaves and roots of HD961 and 9311 when treated with Uniconazole compared to salt stress, and the increase in antioxidant enzyme activities was greater in the leaves and roots of 9311. This indicates that the application of Uniconazole increased antioxidant activity, effectively reduced ROS levels, and protected the plant from the damaging effects of salt-induced oxidative stress (Mahmood et al., 2021; Parveen et al., 2019). This finding is consistent with previous studies on the effectiveness of Uniconazole in alleviating drought stress in maize (Ahmad et al., 2018b). Meanwhile, our results were consistent with the findings of Zhang Panpan et al., demonstrating that foliar application of Uniconazole under salt stress can substantially boost the activity of antioxidant enzymes in plants, decrease cell membrane damage, preserve membrane system stability, and improve plant resilience to adverse conditions (Zhang et al., 2020). Yang found that application of Uniconazole enhanced cold tolerance in rice by increasing antioxidant enzyme activities (Yu et al., 2003), which is similar to the results of the present study. In addition, the study’s findings revealed that the levels of antioxidant enzymes (APX and CAT) in the root system of the salt-tolerant rice variety HD961 were higher compared to those in the salt-sensitive rice variety 9311 after the application of Uniconazole under salt stress. This difference may be attributed to the varying responses of different salt-tolerant rice varieties to stress. Previous studies have indicated that rice varieties with robust stress resistance exhibit higher antioxidant enzyme activities than sensitive varieties (Guo et al., 2006). On the contrary, the activities of antioxidant enzymes (APX and CAT) in the leaves of salt-tolerant rice HD961 were lower than those of the salt-sensitive rice variety 9311, possibly due to the differences in plant types among rice varieties. The above results show that Uniconazole effectively activates the antioxidant system of rice under salt stress, balances the ROS content in plants, reduces the degradation of photosynthetic pigments, and maintains the photosynthetic function of plant leaves, thereby increasing the yield. However, POD activity was higher in roots than in leaves, and APX and CAT activities were higher in leaves than in roots, which may be due to the fact that the roots were more sensitive to the response of POD activity under salt stress. The results show that exogenous Uniconazole can effectively improve the antioxidant efficiency of rice under salt stress, inhibit ROS accumulation, reduce membrane lipid peroxidation, and reduce the toxic effects of salt stress on rice. This method could provide some reference for the cultivation of rice in saline-alkali soil.

Abiotic stresses, such as drought, salinity, heavy metals, and temperature, have antagonistic effects on plant growth and development, resulting in an overall decline in plant yield (Khan et al., 2021), which poses a sever risk to global food supply. In contrast, the number of panicles, grain number per panicle, 1000-grain weight, and yield of rice were significantly decreased under salt stress, with the number of panicles being one of the primary factors contributing to yield reduction under salt stress. During the entire life cycle of rice, the booting stage is a critical period that determines the development or degradation of spikelets. Previous studies have shown that stress during the booting stage of rice can impact spike and yield traits, such as causing a significant decrease in panicle length, branch number, and grain size, which ultimately affects the 1000-grain weight and yield of rice (Gerona et al., 2019; Wang et al., 2020). In this study, salt stress significantly reduced the yield components of HD961, such as panicle length, panicle number per hole, filled grain number, 1000-grain weight, and yield per plant. The reduction in plant yield under salt stress is due to a decrease in the conversion of soluble sugar or starch content in rice spikelets and a decline in starch synthase activity during grain development (Abdullah et al., 2001). Salt stress may also inhibit the rice root system from suppressing water uptake at the reproductive stage, leading to disruptions in physiological processes that result in suboptimal production and distribution of assimilates (Nasrudin et al., 2022), ultimately reducing production. In contrast, foliar application of Uniconazole under salt stress increased the yield components related to the rice variety HD961. Uniconazole may enhance the growth of rice spikelets under salt stress, thereby alleviating the yield loss caused by salt stress. Meanwhile, Uniconazole increased the photosynthetic rate of HD961 under salt stress, and it has been demonstrated that the yield of rice can be increased by enhancing the net photosynthetic rate (*P_n_*) (Ainsworth and Long, 2021; Long et al., 2006). Our results are consistent with earlier studies, both of which indicate that the application of Uniconazole accelerates salt and water uptake by the plant, thereby increasing plant yield and salt tolerance (Hala and Eleiwa, 2008). As shown in **Figures 8A–C**, Uniconazole treatment significantly improved grain yield and panicle traits under salt stress. A possible reason for the higher grain yield in Uniconazole treatment is the reduction in the rate of collapse. Another possibility is that Uniconazole can improve the growth and development of plants under stress and increase yield by enhancing the antioxidant defense system (Ahmad et al., 2018a). Furthermore, correlation analysis revealed that rice yield and yield-related characteristics were positively linked with photosynthetic indicators and antioxidant enzyme activities (**Figure 10**). However, exogenous spraying of Uniconazole did not improve the seed setting rate of HD961 under salt stress, but the grain yield per plant and the number of panicles per hole were significantly increased compared to salt stress. This suggests that Uniconazole may improve yield by increasing the number of effective tillers. In the present study, application of Uniconazole significantly increased yield and related yield components, indicating that Uniconazole has the potential to protect the development of rice spike differentiation and increase yield.

## 5 Conclusion

**Figure 11:**
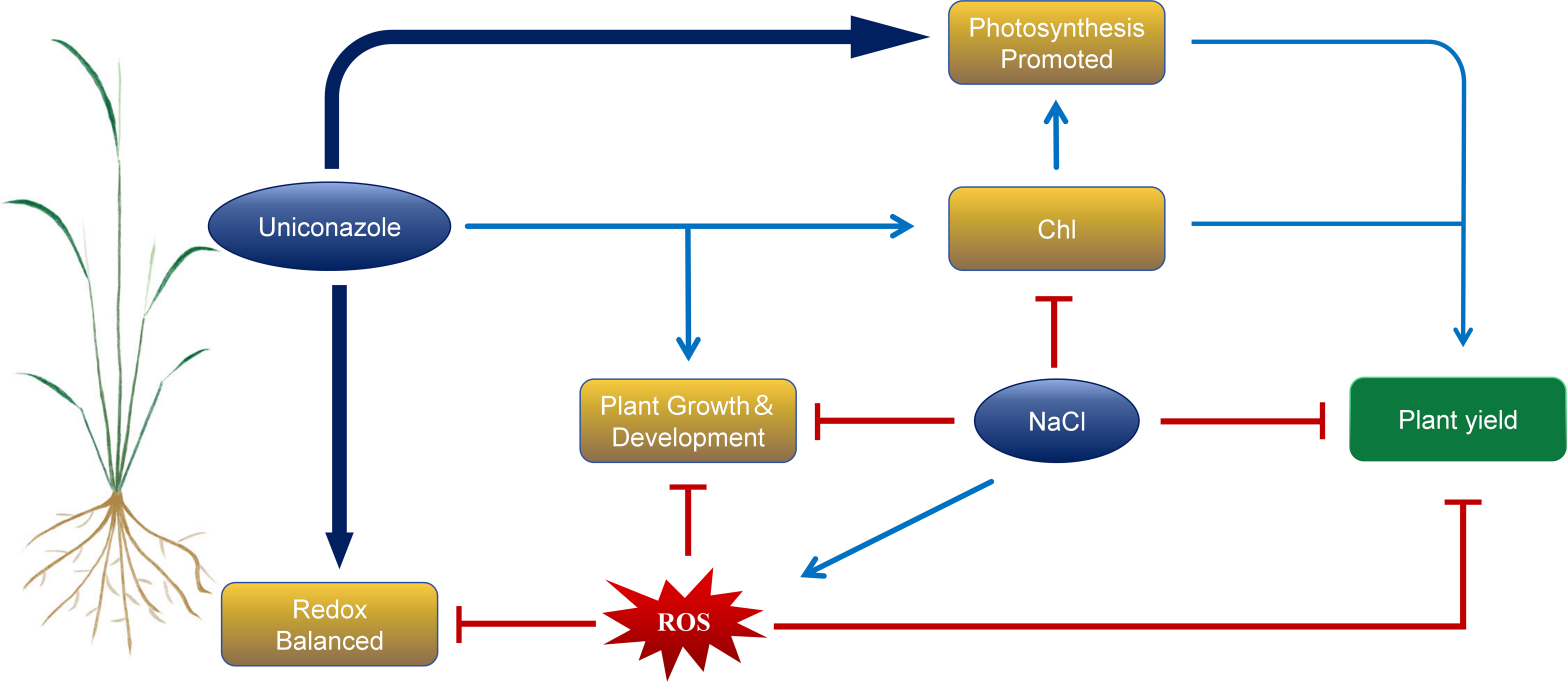
Response mechanism model of Uniconazole-induced rice under salt stress. The red icon indicates inhibition, while the blue arrow indicates promotion.

In summary, salt stress significantly inhibited the plant height, stem diameter, leaf area, and root growth, such as root length, root surface area, root volume, root average diameter, root dry weight, and fresh weight in the seedling stage of two rice varieties, HD961 and 9311. At the same time, salt stress reduces the antioxidant capacity of plants and broke the balance of reactive oxygen species, resulting in a decrease in biomass. However, exogenous application of Uniconazole can regulate the morphological, physiological, and biochemical parameters, enhancing the salt tolerance in rice under salt stress. Uniconazole enhances the activities of antioxidant enzymes such as POD, APX and CAT in rice to cope with the oxidative damage caused by salt stress. At the same time, the chlorophyll content of two rice varieties was increased, enhancing photosynthesis, thereby delaying plant senescence and sustaining normal plant growth. Notably, Uniconazole was more effective in regulating the salt-sensitive rice variety 9311. The results of this study showed that exogenous foliar spraying of Uniconazole under salt stress could alleviate the inhibition of aboveground growth and development of rice, promote the growth of root system, increase the content of photosynthetic pigment, enhancing photosynthesis, and improve the salt tolerance and yield of rice (**Figure 11**). This article is based on pot experiments, so it has certain limitations. However, it also offers insights on the use of Uniconazole to improve the salt tolerance of rice seedlings on coastal beaches. This can help ensure the relatively stable growth of rice in high-salt areas to a certain extent.

## Data Availability

The original contributions presented in the study are included in the article/supplementary material. Further inquiries can be directed to the corresponding authors.
